# Inhibitory effect of a cholecystokinin antagonist on pancreatic carcinogenesis after pancreatobiliary diversion.

**DOI:** 10.1038/bjc.1993.123

**Published:** 1993-04

**Authors:** P. Watanapa, B. Flaks, H. Oztas, P. H. Deprez, J. Calam, R. C. Williamson

**Affiliations:** Department of Surgery, Royal Postgraduate Medical School, Hammersmith Hospital, London, UK.

## Abstract

The role of cholecystokinin (CCK) has been explored in pancreatic carcinogenesis following pancreatobiliary diversion (PBD), using the specific CCK receptor antagonist CR-1409. Male Wistar rats (n = 80) weighing 70-100 g were given weekly i.p. injections of azaserine (30 mg kg-1 week-1) for 3 consecutive weeks. One week later animals were randomised to receive either PBD or sham PBD and thereafter to receive s.c. injections of either saline or CR-1409 (10 mg kg-1 day-1, 5 days a week). Six months after operation surviving rats were killed as follows: sham + saline 20, PBD + saline 19, sham + CR-1409 14, PBD + CR-1409 11. Cardiac blood was taken for CCK assay and the pancreas was excised for wet weight measurement and quantitative estimation of atypical acinar cell foci (AACF), the precursor of carcinoma. PBD reduced median body weight (3-20% less than shams) but trebled the absolute and relative pancreatic weights (P < 0.001). CR-1409 blunted this adaptive response to PBD, reducing absolute pancreatic weight by 35% (P < 0.005). PBD quadrupled circulating CCK concentrations, regardless of the antagonist treatment. Acidophilic AACF occurred only in rats with PBD. CR-1409 markedly reduced the number of observed acidophilic AACF by 90% (P < 0.001) and the number of foci per pancreas by 93% (P < 0.001). Moreover, CR-1409 reduced the mean focal diameter of each lesion by 18% (P < 0.005), the mean focal volume by 58% (P < 0.05) and the percentage of pancreas occupied by acidophilic foci by 95% (P < 0.001). PBD enhances pancreatic carcinogenesis by causing hypercholecystokininaemia, and CR-1409 largely inhibits this enhancement.


Br. J. Cancer (1993), 67, 663–667 © Macmillan Press Ltd., 1993

### Inhibitory effect of a cholecystokinin antagonist on pancreatic carcinogenesis after pancreatobiliary diversion

P. Watanapa[1,*], B. Flaks[3], H. Oztas[3], P.H. Deprez[2], J. Calam[2] & R.C.N. Williamson[1]

*Departments of [1]Surgery and [2]Medicine, Royal Postgraduate Medical School, Hammersmith Hospital, Du Cane Road, London W12 0NN; [3]Pathology Division, Environmental Toxicology Centre, Churchill Building, Langford House, Langford BS18 7DU, UK.*

**Summary** The role of cholecystokinin (CCK) has been explored in pancreatic carcinogenesis following pancreatobiliary diversion (PBD), using the specific CCK receptor antagonist CR-1409. Male Wistar rats ($n = 80$) weighing 70–100 g were given weekly i.p. injections of azaserine (30 mg $kg^{-1}$ $week^{-1}$) for 3 consecutive weeks. One week later animals were randomised to receive either PBD or sham PBD and thereafter to receive s.c. injections of either saline or CR-1409 (10 mg $kg^{-1}$ $day^{-1}$, 5 days a week). Six months after operation surviving rats were killed as follows: sham + saline 20, PBD + saline 19, sham + CR-1409 14, PBD + CR-1409 11. Cardiac blood was taken for CCK assay and the pancreas was excised for wet weight measurement and quantitative estimation of atypical acinar cell foci (AACF), the precursor of carcinoma. PBD reduced median body weight (3–20% less than shams) but trebled the absolute and relative pancreatic weights ($P < 0.001$). CR-1409 blunted this adaptive response to PBD, reducing absolute pancreatic weight by 35% ($P < 0.005$). PBD quadrupled circulating CCK concentrations, regardless of the antagonist treatment. Acidophilic AACF occurred only in rats with PBD. CR-1409 markedly reduced the number of observed acidophilic AACF by 90% ($P < 0.001$) and the number of foci per pancreas by 93% ($P < 0.001$). Moreover, CR-1409 reduced the mean focal diameter of each lesion by 18% ($P < 0.005$), the mean focal volume by 58% ($P < 0.05$) and the percentage of pancreas occupied by acidophilic foci by 95% ($P < 0.001$). PBD enhances pancreatic carcinogenesis by causing hypercholecystokininaemia, and CR-1409 largely inhibits this enhancement.

The growth of experimental pancreatic tumours is regulated by several hormones and growth factors, not only gastrointestinal peptides (Townsend *et al.*, 1989), but also sex hormones (Longnecker & Sumi, 1990), luteinising hormone-releasing hormone (LHRH) (Redding & Schally, 1984), glucocorticoids (Benz *et al.*, 1986) and epidermal growth factor (Malt *et al.*, 1987). Among the gastrointestinal peptides, cholecystokinin (CCK) and its analogues are of particular importance. In the rat and hamster, exogenous CCK and the related substance caerulein stimulate maximal pancreatic secretion and pancreatic growth when administered s.c. for 6 weeks (Barrowman & Mayston, 1974; Dembinski & Johnson, 1980). Measures designed to increase circulating CCK levels, such as dietary administration of trypsin inhibitors or injections of cholecystokinin, appear to enhance pancreatic carcinogenesis in azaserine-treated rats (Lhoste *et al.*, 1988; Douglas *et al.*, 1989), but similar studies in hamsters have given inconsistent results (Andrén-Sandberg *et al.*, 1984; Howatson & Carter, 1985; Pour *et al.*, 1988).

Diversion of bile and pancreatic secretions to the mid small bowel causes pancreatic hyperplasia in rats (Miazzi *et al.*, 1987; Watanapa *et al.*, 1991). The associated hypercholecystokininaemia plus the inhibitory effect of the CCK anatagonist CR-1409 indicate the pivotal role of CCK in the adaptive response to pancreatobiliary diversion (PBD) (Watanapa *et al.*, 1991). Our long-term study in rats showed that both PBD and massive small bowel resection will stimulate pancreatic growth, but only PBD enhances pancreatic carcinogenesis (Stewart *et al.*, 1991); CCK levels were not measured in this experiment.

The present study was designed to test the hypothesis that hypercholecystokininaemia explains the enhancing effect of PBD on pancreatic carcinogenesis induced by azaserine in rats. We used quantitative estimation of atypical acinar cell foci (AACF) as the index of neoplastic change, in line with others (Roebuck *et al.*, 1984; Sumi *et al.*, 1989), and the specific CCK antagonist CR-1409 to inhibit the response.

### Methods

### *Animal design*

Male Wistar rats ($n = 80$) aged 4 weeks and weighing 70–100 g were housed in groups of eight and later of five in animal quarters with a 12 h day/night cycle. Standard pelleted rat food (Paterson and the Christopher Hill Group, Porton – diet PRD) and water were freely available. After 1 week of acclimatisation, all animals received weekly i.p. injections of azaserine for 3 weeks (see below). One week after the end of this course, animals were randomised to receive either pancreatobiliary diversion (PBD) or sham PBD, comprising triple small bowel transection and resuture. PBD involved transposition of 50 cm proximal small bowel to lie between the pylorus and duodenal papilla, whereas in shams the small bowel was divided immediately distal to the pylorus, at the duodenojejunal junction and again at the level of the mid-small bowel. Operations were carried out under light ether anaesthesia. A continuous 6/0 silk suture was used for anastomoses.

Immediately after the operation, half the animals in each group were further randomised to receive either CR-1409 (10 mg $kg^{-1}$ $day^{-1}$) or saline (2.5 ml $kg^{-1}$) by daily subcutaneous injection, 5 days per week. CR-1409 was dissolved in distilled water and brought to pH 9 by 0.01 N NaOH to give a 0.4% solution. Food was reintroduced 12 h postoperatively. Six months after operation, blood samples for CCK assay were obtained by direct cardiac puncture after overnight fasting; rats were then killed by exsanguination. The pancreas was excised and trimmed free of adherent fat and lymph nodes. The wet weight of each gland was recorded before fixation in 10% buffered neutral formalin. Before immersion in the fixative solution, each pancreas was spread out on a piece of porous paper to ensure the maximal transectional area for subsequent sectioning.

Correspondence: R.C.N. Williamson, Department of Surgery, Hammersmith Hospital, RPMS, Du Cane Road, London W12 0NN, UK.
*Present address: Department of Surgery, Siriraj Hospital, Bangkok 10700, Thailand.
Received 19 March 1992; and in revised form 9 July 1992.

*Carcinogen*

Azaserine (Sigma Chemical Company, UK) was dissolved in 0.9% NaCl and was administered by weekly i.p. injection into each rat for 3 consecutive weeks. The dosage regime was 30 mg $kg^{-1}$ $week^{-1}$, giving a total dose of 90 mg $kg^{-1}$.

*CCK assay*

Plasma CCK peptides were extracted from cardiac blood samples with C18 'SepPak' (Waters, Harrow, UK) (Eysellein *et al.*, 1987), and the eluates were dried by centrifugal evaporation (Savant, Farmingdale, NY, USA). CCK was measured by a specific radioimmunoassay based on antiserum $A_2$, raised by immunising a rabbit with natural porcine CCK-33 (donated by Professors V. Mutt and S.R. Bloom). Antiserum $A_2$ (1:60,000) was incubated at 4°C for 3 days with standard CCK-8 or with plama samples plus CCK-8 tracer labelled with $^{125}$iodine (1,000 c.p.m., Amersham, UK) in 0.05 mol $l^{-1}$ sodium phosphate buffer (pH 7.4) containing 0.25% gelatin and 0.01% mol $l^{-1}$ EDTA. Free and bound tracer were separated by the addition of 6% (weight/volume) dextran. The concentrations of pure peptides that produced half-maximal inhibition of binding of tracer to $A_2$ were 2.0 pmol $l^{-1}$ for CCK-8, 2.4 pmol $l^{-1}$ for CCK-33 and 2.2 nmol $l^{-1}$ for gastrin 17. The coefficient of variation within assays was 8.2% and between assays 12.8%. The sensitivity of the assay (defined as minimal amount of CCK-8 that could be distinguished from zero with 95% confidence) was 0.2 pmol, and the recovery of CCK-8 and CCK-33 through the SepPak and assay procedure was 79%.

*Quantitative estimation of AACF*

Histological sections (5 μm) of the whole pancreas were stained with haematoxylin and eosin, coded and scrutinised 'blind' by light microscopy. The two observers evaluated each section together and did not know what treatment each animal had received. The atypical acinar cell foci (AACF) were readily identified and classified as acidophilic or basophilic according to established criteria (Rao *et al.*, 1982). The total area of exocrine pancreatic tissue was measured directly in a single histological secion from each pancreas by means of a VIDS III video image analyser (Analytical Measuring Systems, Cambridge). The same instrument was used to count acidophilic and basophilic AACF and to measure their transectional area. Data were processed numerically by the Volugen computer package (InfoResearch Int., Bristol), using an algorithm based on that of Campbell *et al.* (1982) and modified by Pugh *et al.* (1983). The actual numerical lower limits adopted were 0.0005 $mm^2$ for basophilic AACF and 0.01 $mm^2$ for the acidophilic variety; these values correspond to those chosen by Roebuck *et al.* (1984). Details of this analysis have been already described in our previous studies (Stewart *et al.*, 1991).

*Statistical analysis*

Student's *t*-test for unpaired data was used for the group analysis of plasma CCK concentrations, since the data were normally distributed. The levels were expressed as means (s.e.m.). Median values and ranges were quoted for body weight, pancreatic weight and quantitative estimation of AACF. Statistical analysis of these parameters was performed using Kruskal-Wallis analysis of variance and the Mann-Whitney U-test.

### Results

*Mortality, body weight and pancreatic weight (Table I)*

There are five early deaths from anaesthetic overdose or anastomotic leakage, one in a sham + CR-1409 rat and four in PBD + CR-1409 rats. Eleven rats died prematurely from instestinal obstruction or extensive granuloma formation, related either to anastomotic leakage or to repeated i.p. injections: one in the PBD + saline group, five in the sham + CR-1409 group and five in the PBD + CR-1409 group. Yields of healthy survivors were as follows: sham + saline 20, PBD + saline 19, sham + CR-1409 14 and PBD + CR-1409 11.

The median body weight of PBD rats was 3–20% less than shams, although the reduction was only significant in the CR-1409 treated groups. Likewise, animals receiving CR-1409 weighed a median 5–22% less than their saline-treated counterparts. Median pancreatic weight was markedly increased by PBD, both absolute weight, which was 173% greater, and relative weight (mg pancreas/100 g body weight), which was 206% greater. The CCK antagonist CR-1409 had differential effects in shams and rats with PBD. In shams it caused modest elevations in pancreatic weight of 38–48%. By contrast, CR-1409 partly inhibited the growth response to PBD, reducing absolute pancreatic weight by 35%, although these rats still had pancreata that were 30–62% heavier than those of their sham counterparts.

The pancreata in several sham and some PBD animals receiving CR-1409 showed extensive degeneration as characterised by patchy necrosis, cellular infiltration and fibrosis. Some pancreatic acinar cells also showed cytoplasmic vacuoles and loss of zymogen granules. These findings were not seen in the saline-treated rats.

*Plasma cholecystokinin (Table II)*

There was a 4-fold increase in circulating CCK levels 6 months after PBD. Administration of CR-1409 had no appreciable effect on plasma hormone levels.

*Quantiative analysis of AACF*

*Acidophilic* AACF, the putative precancerous lesions, were only seen in rats receiving PBD (Table III). CR-1409 substantially reduced the observed transectional data, so that the number of AACF per $cm^2$ pancreas was only one tenth of those seen in controls given saline. Quantitative stereological analysis of tissue sections confirmed the inhibitory effect of CR-1409. Thus the number of acidophilic AACF per $cm^3$ of pancreas was markedly reduced (3.00 *vs* 33.93), as was the total number of lesions per pancreas (5.73 *vs* 77.10). The median diameter of each focus was 18% less and the volume was 58% less. Consequently CR-1409 reduced the percentage of the pancreatic volume occupied by acidophilic foci from 2.54% to 0.12%.

**Table I** Body weight, absolute and relative pancreatic weight

| | *Sham + saline* (*n* = *20*) | *Sham + CR-1409* (*n* = *14*) | *PBD + saline* (*n* = *19*) | *PBD + CR-1409* (*n* = *11*) |
|---|---|---|---|---|
| Body weight (g) | 523.1 | 483.0[e] | 516.0 | [d]404.0[b] |
| | (440.0–551.0) | (303.0–547.0) | (310.0–563.0) | (275.0–576.0) |
| Absolute pancreatic weight (mg) | 882.0 | 1165.0[a] | 2310.0[a] | [d]1510.0[b] |
| | (750.0–1100.0) | (950.0–1650.0) | (1250.0–3850.0) | (950.0–2400.0) |
| Relative pancreatic weight (mg/100 g body weight) | 168.6 | 246.6[a] | 509.8[a] | 398.4[c] |
| | (143.1–206.4) | (192.7–320.4) | (241.3–808.2) | (260.4–583.3) |

Values are median (range). Significance *vs* sham + saline group: [a]$P<0.001$; [e]$P<0.05$. Significance *vs* sham + CR-1409 group: [b]$P<0.05$; [c]$P<0.001$. Significance *vs* PBD + saline group: [d]$P<0.05$.

**Table II** Plasma cholecystokinin concentration ($pmol\ l^{-1}$) in rats with pancreatobiliary diversion (PBD) or sham PBD

| | *Sham + saline (n = 20)* | *Sham + CR-1409 (n = 14)* | *PBD + saline (n = 19)* | *PBD + CR-1409 (n = 11)* |
|---|---|---|---|---|
| Plasma CCK ($pmol\ l^{-1}$) | 1.81 (0.13) | 2.93 (0.43) | 8.23 (0.87)[a] | 10.95 (1.16)[a] |

Values are means (s.e.m.). Significance *vs* corresponding sham group: [a]$P<0.001$.

**Table III** Quantitative analysis of acidophilic atypical acinar cell foci (AACF) in the pancreas of azaserine-treated rats with pancreatobiliary diversion (PBD) or sham PBD

| | *Sham + saline (n = 20)* | *Sham + CR-1409 (n = 14)* | *PBD + saline (n = 19)* | *PBD + CR-1409 (n = 11)* |
|---|---|---|---|---|
| No. of AACF $cm^{-2}$ | 0.00 | 0.00 | 3.44[a] (0.69–11.47) | [d]0.34[a] (0.00–3.03) |
| No. of AACF $cm^{-3}$ | 0.00 | 0.00 | 33.93[a] (6.56–117.32) | [d]3.00[a] (0.00–34.61) |
| No. of AACF/pancreas | 0.00 | 0.00 | 77.10[a] (10.50–352.95) | [d]5.73[a] (0.00–60.56) |
| Mean focal diameter (μm) | 0.00 | 0.00 | 1064.80[a] (719.90–1637.00) | [c]871.00[a] (0.00–1207.74) |
| Mean focal volume ($mm^3 \times 100$) | 0.00 | 0.00 | 51.45[a] (13.20–173.11) | [b]21.79[a] (0.00–62.84) |
| Volume as % of pancreas | 0.00 | 0.00 | 2.54[a] (0.25–6.60) | [d]0.12[a] (0.00–1.13) |

Values are medians (range). Only foci that are larger than 0.01 $mm^2$ are counted. Significance *vs* sham + saline group: [a]$P<0.001$. Significance *vs* PBD + saline group: [b]$P<0.05$; [c]$P<0.005$; [d]$P<0.001$.

By contrast, *basophilic* AACF occurred mainly in the sham animals (Table IV), and the CCK antagonist had no consistent effect on their development. Although saline-treated rats had more lesions than those receiving CR-1409, the size of each focus was smaller, so that a similar percentage of the pancreas was occupied by basophilic foci.

### Discussion

Our data confirm the potent effect of pancreatobiliary diversion in enhancing experimental pancreatic carcinogenesis and strongly support the hypothesis that hyperchlecystokininaemia is the key intermediary (Miazza *et al.*, 1987; Stewart *et al.*, 1991; Watanapa *et al.*, 1991). Not only were circulating CCK levels elevated after PBD, but in addition the specific antagonist CR-1409 sharply diminished the number of acidophilic AACF. These foci are now well established as the precursors of pancreatic carcinoma in this model, whereas basophilic foci appear to be of little importance. The acidophilic foci show considerable growth potential with a mitotic index (2.75) which greatly exceeds that of basophilic foci (0.125) or normal pancreas (zero) (Scarpelli *et al.*, 1984). Using [$^3$H] thymidine incorporation and autoradiography, Rao and colleagues (1982) demonstrated much greater proliferative capacity of cells in acidophilic foci than basophilic foci (23.2 *vs* 1.2 labelled nuclei per 1,000 cells). Although fewer than 1% of these acidophilic AACF progress to become neoplasms (Longnecker *et al.*, 1979), the number and size of acidophilic AACF seem to correlate positively with the incidence of carcinoma (Roebuck & Longnecker, 1977; Longnecker *et al.*, 1981).

The finding that PBD increases pancreatic weight as well as promoting carcinogenesis suggests that in the pancreas, as in the large intestine (Bristol *et al.*, 1984), hyperplasia precedes and predisposes to neoplasia. Measurements of relative and absolute wet weights are relatively crude indices of pancreatic adaptation, but the necessity of measuring AACF precluded any more sophisticated tests in the present experiment. We have previously shown that PBD causes pancreatic hyperplasia, with increased contents of RNA and DNA and increased cytokinetic indices 4 to 14 days after operation (Watanapa *et al.*, 1991). As in the present experiment, CR-1409 inhibited the growth-promoting effect of PBD without

**Table IV** Quantitative analysis of basophilic atypical acinar cell foci (AACF) in the pancreas of azaserine-treated rats with pancreatobiliary diversion (PBD) or sham PBD

| | *Sham + saline (n = 20)* | *Sham + CR-1409 (n = 14)* | *PBD + saline (n = 19)* | *PBD + CR-1409 (n = 11)* |
|---|---|---|---|---|
| No. of AACF $cm^{-2}$ | 1.42 (0.38–6.60) | 1.16 (0.00–3.93) | 0.00[a] (0.00–0.41) | [c]0.00[a] (0.00–0.24) |
| No. of AACF $cm^{-3}$ | 43.10 (11.81–246.66) | 28.26 (0.00–93.23) | 0.00[a] (0.00–11.91) | [c]0.00[a] (0.00–9.01) |
| No. of AACF/pancreas | 34.50 (11.22–184.99) | 31.15 (0.00–104.42) | 0.00[a] (0.00–36.16) | [c]0.00[a] (0.00–13.61) |
| Mean focal diameter (μm) | 327.24 (268.22–434.58) | 394.10 (0.00–709.07) | 0.00[a] (0.00–379.93) | [b]0.00[a] (0.00–467.91) |
| Mean focal volume ($mm^3 \times 100$) | 1.33 (0.54–2.91) | 2.24 (0.00–19.77) | 0.00[a] (0.00–2.68) | [b]0.00[a] (0.00–3.77) |
| Volume as % of pancreas | 0.08 (0.01–0.32) | 0.07 (0.00–0.36) | 0.00[a] (0.00–0.03) | [c]0.00[a] (0.00–0.02) |

Values are medians (range). Only foci that are larger than 0.0005 $mm^2$ are counted. Significance *vs* sham + saline group: [a]$P<0.001$. Significance *vs* CR-1409 group: [b]$P<0.05$; [c]$P<0.005$.

affecting plasma CCK levels, showing that the drug probably acts as a receptor antagonist (Leung et al., 1986). Our preliminary ultrastructural data indicate that CR-1409 causes marked vacuolation in the cytoplasm of pancreatic acinar cells, suggesting a direct toxic effect (Sarraf et al., 1990). The inflammatory changes seen in shams with CR-1409 may explain the increased pancreatic weight in these animals, while in rats with PBD this effect was overcome by the growth stimulatory response. In line with Douglas and colleagues (1989), our finding that CR-1409 reduced both pancreatic weight and the number of AACF supports the association between hyperplasia and neoplasia. To the contrary, one stimulus (massive enterectomy) can cause pancreatic growth without promoting cancer (Stewart et al., 1991), while another (exogenous caerulein) can cause the reverse (Lhoste & Longnecker, 1987). Thus the co-carcinogenic effect of PBD might be partly independent of its growth-promoting effect and could reflect a direct action of CCK on malignant transformation of the exocrine pancreas.

The development of AACF is influenced by the age of the rats at the onset of treatment (Longnecker et al., 1977). The low yield rate of acidophilic AACF in this experiment may reflect the greater age of animals used compared to other studies (4 weeks vs 19 days and 14 days) (Roebuck et al., 1985; Douglas et al., 1989). Among saline-treated animals, the increased yield of basophilic foci in controls (as opposed to those with PBD) is of doubtful relevance since most modulators of the postinitiation phase of pancreatic carcinogenesis have little effect on these lesions (Roebuck et al., 1985).

There are two possible explanations for the hypercholecystokininaemia that follows PBD (Miazza et al., 1987; Watanapa et al., 1991): (1) increased CCK secretion, as diversion of pancreatobiliary secretions away from the transposed jejunum suppresses the normal negative feedback mechanism and (2) increased CCK synthesis, if the jejunal hyperplasia that follows PBD (Miazza et al., 1982; Hosomi et al., 1987) involves the enteroendocrine cells. We have previously found 2-fold elevations in circulating CCK 7–14 days after PBD (Watanapa et al., 1991), and we now find 4-fold elevations 6 months later. The progressive rise in CCK may argue more for an increased synthesis of the hormone than for perpetual interruption of feedback inhibition.

We thank the Royal Postgraduate Medical School and the Hammersmith and Queen Charlotte's Special Health Authority for supporting this research. The CCK anatagonist CR-1409 was kindly provided by Dr L. Rovati of Rotta Research Laboratories (Milan, Italy).

### References

ANDRÉN-SANDBERG, Å., DAWISKIBA, S. & IHSE, I. (1984). Studies of the effect of cerulein administration on experimental pancreatic carcinogenesis. *Scand. J. Gastroenterol.*, **19**, 122–128.

BARROWMAN, J.A. & MAYSTON, P.D. (1974). Proceedings: the trophic influence of cholecystokinin on the rat pancreas. *J. Physiol.*, **238**, 73p–75p.

BENZ, C., HOLLANDER, C. & MILLER, B. (1986). Endocrine-responsive pancreatic carcinoma: steroid binding and cytotoxicity studies in human tumor cell lines. *Cancer Res.*, **46**, 2276–2281.

BRISTOL, J.B., WELLS, M. & WILLIAMSON, R.C.N. (1984). Adaptation to jejunoileal bypass promotes experimental colorectal carcinogenesis. *Br. J. Surg.*, **71**, 123–126.

CAMPBELL, H.A., PITOT, H.C., POTTER, V.R. & LAISHES, B.A. (1982). Application of quantitative stereology to the evaluation of enzyme altered foci in rat liver. *Cancer Res.*, **42**, 465–472.

DEMBINSKI, A.B. & JOHNSON, L.R. (1980). Stimulation of pancreatic growth by secretin, caerulein and pentagastrin. *Endocrinology*, **106**, 323–328.

DOUGLAS, B.R., WOUTERSEN, R.A., JANSEN, J.B.M.J., DE JONG, A.J.L., ROVATI, L.C. & LAMERS, C.B.H.W. (1989). Modulation by CR-1409 (lorglumide), a cholecystokinin receptor antagonist, of trypsin inhibitor-enhanced growth of azaserine-induced putative preneoplastic lesions in rat pancreas. *Cancer Res.*, **49**,2438–2441.

EYSELLEIN, V.E., EBERLEIN, G.A., HESSE, W.H., SINGER, M.V., GOEBELL, H. & REEVE, J.R. Jr. (1987). Cholecystokinin-58 is the major circulating form of cholecystokinin in canine blood. *J. Biol. Chem.*, **262**, 214–217.

HOSOMI, M., LIRUSSI, F., STACE, N.H., VAJA, S., MURPHY, G.M. & DOWLING, R.H. (1987). Mucosal polyamine profile in normal and adapting (hypo and hyperplasia) intestine: effects of DFMO treatment. *Gut*, **28** (suppl 1), 103–107.

HOWATSON, A.G. & CARTER, D.C. (1985). Pancreatic carcinogenesis-enhanced by cholecystokinin in the hamster-nitrosamine model. *Br. J. Cancer*, **51**, 107–114.

LEUNG, Y.K., LEE, P.C. & LEBENTHAL, E. (1986). Maturation of cholecystokinin receptors in pancreatic acini of rats. *Am. J. Physiol.*, **250**, G594–G597.

LHOSTE, E.F. & LONGNECKER, D.S. (1987). Effect of bombesin and caerulein on early stages of carcinogenesis induced by azaserine in the rat pancreas. *Cancer Res.*, **47**, 3273–3277.

LHOSTE, E.F, ROEBUCK, B.D. & LONGNECKER, D.S. (1988). Stimulation of the growth of azaserine-induced nodules in the rat pancreas by dietary camostate (FOY-305). *Carcinogenesis*, **9**, 901–906.

LONGNECKER, D.S., FRENCH, J., HYDE, E., LILJA, H.S. & YAGER, J.D. Jr. (1977). Effect of age on nodule induction by azaserine and DNA synthesis in rat pancreas. *J. Natl Cancer Inst.*, **58**, 1769–1775.

LONGNECKER, D.S., LILJA, H.S., FRENCH, J.I., KUHLMANN, E. & NOLL, W.W. (1979). Transplantation of azaserine-induced carcinomas of pancreas in rats. *Cancer Lett.*, **7**, 197–202.

LONGNECKER, D.S., ROEBUCK, B.D., YAGER, J.D. Jr., LILJA, H.S. & SIEGMUND, B. (1981). Pancreatic carcinoma in azaserine-treated rats: induction, classification, and dieatary modulation of incidence *Cancer*, **47**, 1562–1572.

LONGNECKER, D.S. & SUMI, C. (1990). Effects of sex steroid hormones on pancreatic cancer in the rat. *Int. J. Pancreatol.*, **7**, 159–165.

MALT, R.A., CHESTER, J.F., GAISSERT, H.A. & ROSS, J.S. (1987). Augmentation of chemically induced pancreatic and bronchial cancers by epidermal growth factor. *Gut*, **28** (Suppl 1), 249–252.

MIAZZI, B.M., VAN HUNG, L., VAJA, S. & DOWLING, R.H. (1982). Effect of pancreatico-biliary diversion (PBD) on jejunal and ileal structure and function in the rat. In *Mechanisms of Intestinal Adaptation*, Robinson, J.W.L., Dowling, R.H. & Riecken, E.-O. (eds) pp. 467–476. MTP press: Lancaster.

MIAZZA, B.M., WIDGREN, S., CHAYVIALLE, J.A., NICOLET, T. & LOIZEAU, E. (1987). Exocrine pancreatic nodules after longterm pancreaticobiliary diversion in rats. An effect of raised CCK plasma concentrations. *Gut*, **28** (Suppl. 1), 269–273.

POUR, P.M., LAWSON, T., HELGESON, S., DONNELLY, T. & STEPAN, K. (1988). Effect of cholecystokinin on pancreatic carcinogenesis in the hamster model. *Carcinogenesis*, **9**, 597–601.

PUGH, T.D., KING, J.H., KOEN, H., NYCHKA, D., CHOVER, J., WAHBA, G., HE, Y. & GOLDFARB, S. (1983). Reliable stereological method for estimating the number of microscopic hepatocellular foci from their transections. *Cancer Res.*, **43**, 1261–1268.

RAO, M.S., UPTON, M.P., SUBBARAO, V. & SCARPELLI, D.G. (1982). Two populations of cells with differing proliferative capacities in atypical acinar cell foci induced by 4-hydroxyaminoquinoline-1-oxide in the rat pancreas. *Lab. Invest.*, **46**, 527–534.

REDDING, T.W. & SCHALLY, A.V. (1984). Inhibition of growth of pancreatic carcinoma in animal models by analogs of hypothalamic hormones. *Proc. Natl Acad. Sci.*, **81**, 248–252.

ROEBUCK, B.D. & LONGNECKER, D.S. (1977). Species and rat strain variation in pancreatic nodule induction by azaserine. *J. Natl Cancer Inst.*, **59**, 1273–1277.

ROEBUCK, B.D., BAUMGARTNER, K.J. & THRON, C.D. (1984). Characterisation of two populations of pancreatic atypical acinar cell foci induced by azaserine in the rat. *Lab. Invest.*, **50**, 141–146.

ROEBUCK, B.D., LONGNECKER, D.S., BAUMGARTNER, K.J. & THRON, C.D. (1985). Carcinogen-induced lesions in the rat pancreas: effects of varying levels of essential fatty acid. *Cancer Res.*, **45**, 5252–5256.

SARRAF, C.E., WATANAPA, P. & ALISON, M.R. (1990). Pancreatic hyperplasia as a result of pancreatobiliary diversion (PBD). *J. Physiol.*, **161**, 351A.

SCARPELLI, D.G., RAO, M.S. & REDDY, J.K. (1984). Studies of pancreatic carcinogenesis in different models. *Environ. Health. Perspect.*, **56**, 219–227.

STEWART, I.D., FLAKS, B., WATANAPA, P., DAVIES, P.W. & WILLIAMSON, R.C.N. (1991). Pancreatobiliary diversion enhances experimental pancreatic carcinogenesis. *Br. J. Cancer*, **63**, 63–66.

SUMI, C., LONGNECKER, D.S., ROEBUCK, B.D. & BRINCK-JOHNSEN, T. (1989). Inhibitory effects of estrogen and castration on the early stage of pancreatic carcinogenesis in Fischer rats treated with azaserine. *Cancer Res.*, **49**, 2332–2336.

TOWNSEND, C.M. Jr., SINGH, P. & THOMPSON, J.C. (1989). Effects of gastrointestinal peptides on gastrointestinal cancer growth. *Gastrointest. Endocrinol.*, **18**, 777–791.

WATANAPA, P., EFA, E.F., BEARDSHALL, K., CALAM, J., SARRAF, C.E., ALISON, M.R. & WILLIAMSON, R.C.N. (1991). Inhibitory effect of a cholecystokinin antagonist on the proliferative response of the pancreas to pancreatobiliary diversion. *Gut*, **32**, 1049–1054.


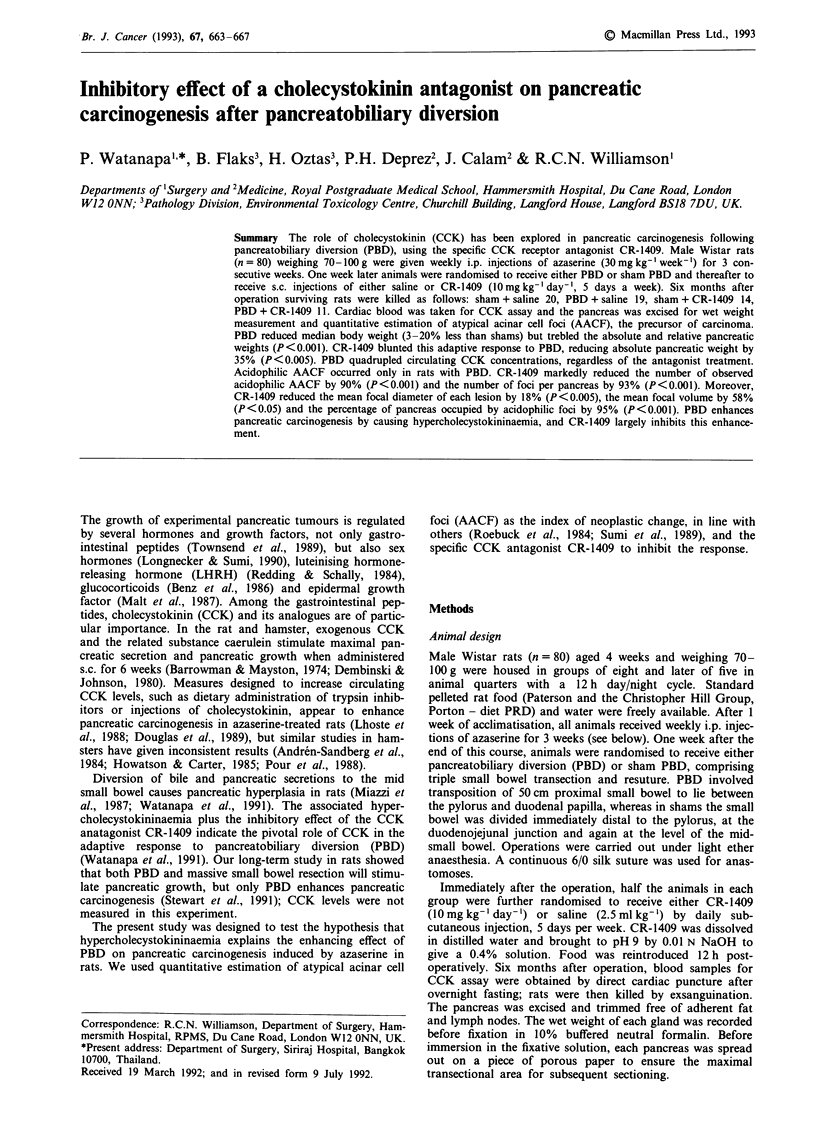

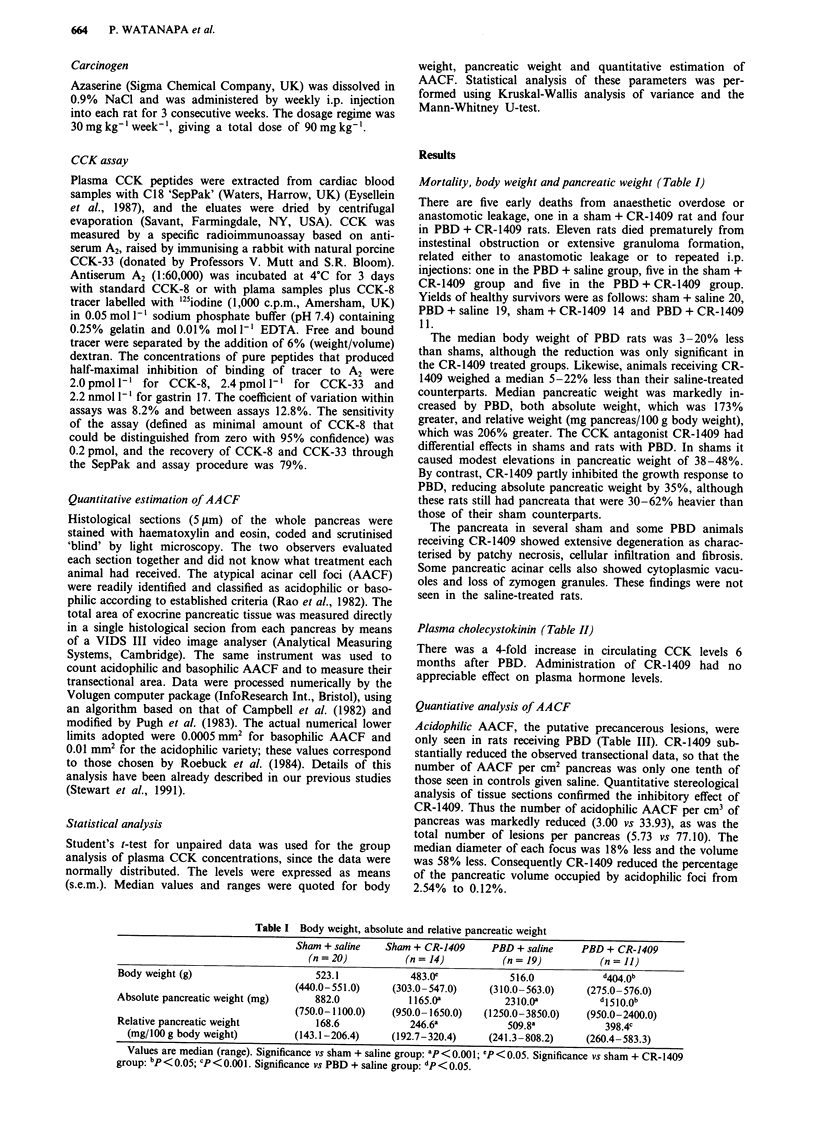

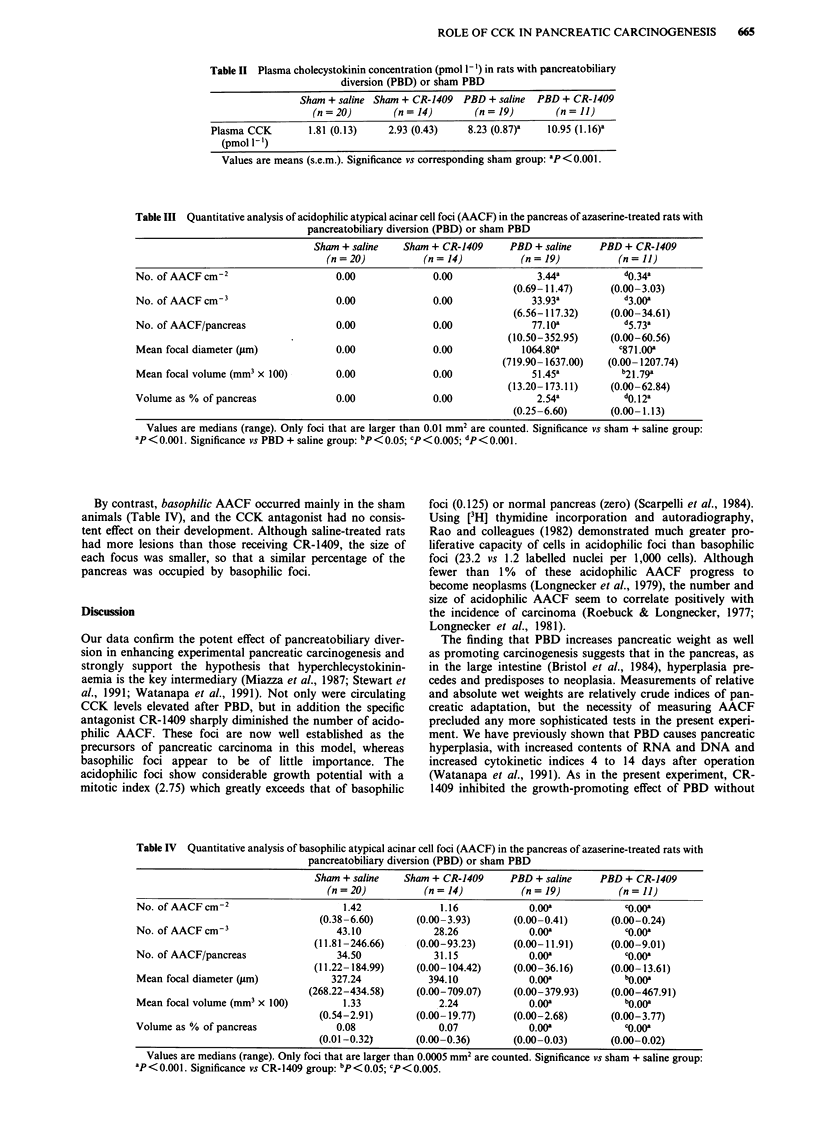

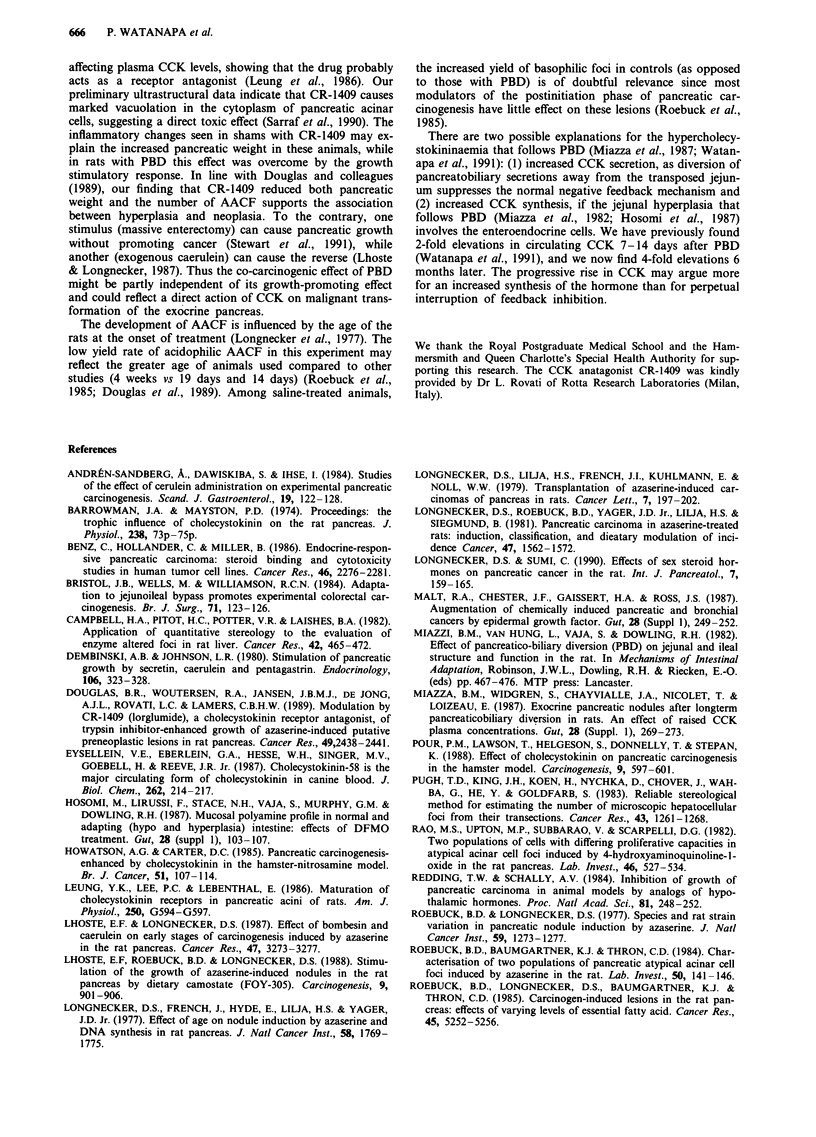

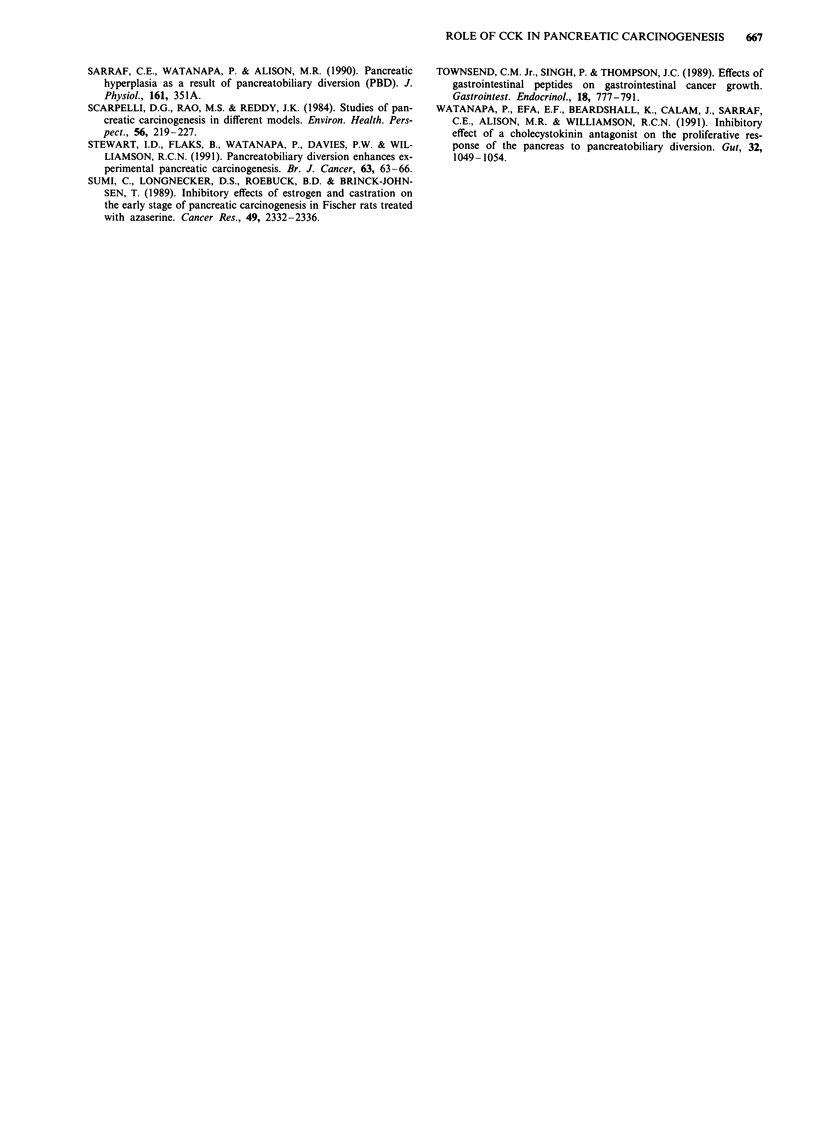


## References

[OCR_00455] Andrén-Sandberg A., Dawiskiba S., Ihse I. (1984). Studies of the effect of cerulein administration on experimental pancreatic carcinogenesis.. Scand J Gastroenterol.

[OCR_00460] Barrowman J. A., Mayston P. D. (1974). Proceedings: The trophic influence of cholecystokinin on the rat pancreas.. J Physiol.

[OCR_00465] Benz C., Hollander C., Miller B. (1986). Endocrine-responsive pancreatic carcinoma: steroid binding and cytotoxicity studies in human tumor cell lines.. Cancer Res.

[OCR_00469] Bristol J. B., Wells M., Williamson R. C. (1984). Adaptation to jejunoileal bypass promotes experimental colorectal carcinogenesis.. Br J Surg.

[OCR_00474] Campbell H. A., Pitot H. C., Potter V. R., Laishes B. A. (1982). Application of quantitative stereology to the evaluation of enzyme-altered foci in rat liver.. Cancer Res.

[OCR_00479] Dembinski A. B., Johnson L. R. (1980). Stimulation of pancreatic growth by secretin, caerulein, and pentagastrin.. Endocrinology.

[OCR_00484] Douglas B. R., Woutersen R. A., Jansen J. B., de Jong A. J., Rovati L. C., Lamers C. B. (1989). Modulation by CR-1409 (lorglumide), a cholecystokinin receptor antagonist, of trypsin inhibitor-enhanced growth of azaserine-induced putative preneoplastic lesions in rat pancreas.. Cancer Res.

[OCR_00490] Eysselein V. E., Eberlein G. A., Hesse W. H., Singer M. V., Goebell H., Reeve J. R. (1987). Cholecystokinin-58 is the major circulating form of cholecystokinin in canine blood.. J Biol Chem.

[OCR_00496] Hosomi M., Lirussi F., Stace N. H., Vaja S., Murphy G. M., Dowling R. H. (1987). Mucosal polyamine profile in normal and adapting (hypo and hyperplastic) intestine: effects of DFMO treatment.. Gut.

[OCR_00502] Howatson A. G., Carter D. C. (1985). Pancreatic carcinogenesis-enhancement by cholecystokinin in the hamster-nitrosamine model.. Br J Cancer.

[OCR_00507] Leung Y. K., Lee P. C., Lebenthal E. (1986). Maturation of cholecystokinin receptors in pancreatic acini of rats.. Am J Physiol.

[OCR_00512] Lhoste E. F., Longnecker D. S. (1987). Effect of bombesin and caerulein on early stages of carcinogenesis induced by azaserine in the rat pancreas.. Cancer Res.

[OCR_00517] Lhoste E. F., Roebuck B. D., Longnecker D. S. (1988). Stimulation of the growth of azaserine-induced nodules in the rat pancreas by dietary camostate (FOY-305).. Carcinogenesis.

[OCR_00523] Longnecker D. S., French J., Hyde E., Lilja H. S., Yager J. D. (1977). Effect of age on nodule induction by azaserine and DNA synthesis in rat pancreas.. J Natl Cancer Inst.

[OCR_00529] Longnecker D. S., Lilja H. S., French J., Kuhlmann E., Noll W. (1979). Transplantation of azaserine-induced carcinomas of pancreas in rats.. Cancer Lett.

[OCR_00534] Longnecker D. S., Roebuck B. D., Yager J. D., Lilja H. S., Siegmund B. (1981). Pancreatic carcinoma in azaserine-treated rats: induction, classification and dietary modulation of incidence.. Cancer.

[OCR_00540] Longnecker D. S., Sumi C. (1990). Effects of sex steroid hormones on pancreatic cancer in the rat.. Int J Pancreatol.

[OCR_00545] Malt R. A., Chester J. F., Gaissert H. A., Ross J. S. (1987). Augmentation of chemically induced pancreatic and bronchial cancers by epidermal growth factor.. Gut.

[OCR_00556] Miazza B. M., Widgren S., Chayvialle J. A., Nicolet T., Loizeau E. (1987). Exocrine pancreatic nodules after longterm pancreaticobiliary diversion in rats. An effect of raised CCK plasma concentrations.. Gut.

[OCR_00562] Pour P. M., Lawson T., Helgeson S., Donnelly T., Stepan K. (1988). Effect of cholecystokinin on pancreatic carcinogenesis in the hamster model.. Carcinogenesis.

[OCR_00569] Pugh T. D., King J. H., Koen H., Nychka D., Chover J., Wahba G., He Y. H., Goldfarb S. (1983). Reliable stereological method for estimating the number of microscopic hepatocellular foci from their transections.. Cancer Res.

[OCR_00573] Rao M. S., Upton M. P., Subbarao V., Scarpelli D. G. (1982). Two populations of cells with differing proliferative capacities in atypical acinar cell foci induced by 4-hydroxyaminoquinoline-1-oxide in the rat pancreas.. Lab Invest.

[OCR_00579] Redding T. W., Schally A. V. (1984). Inhibition of growth of pancreatic carcinomas in animal models by analogs of hypothalamic hormones.. Proc Natl Acad Sci U S A.

[OCR_00589] Roebuck B. D., Baumgartner K. J., Thron C. D. (1984). Characterization of two populations of pancreatic atypical acinar cell foci induced by azaserine in the rat.. Lab Invest.

[OCR_00593] Roebuck B. D., Longnecker D. S., Baumgartner K. J., Thron C. D. (1985). Carcinogen-induced lesions in the rat pancreas: effects of varying levels of essential fatty acid.. Cancer Res.

[OCR_00584] Roebuck B. D., Longnecker D. S. (1977). Species and rat strain variation in pancreatic nodule induction by azaserine.. J Natl Cancer Inst.

[OCR_00606] Scarpelli D. G., Rao M. S., Reddy J. K. (1984). Studies of pancreatic carcinogenesis in different animal models.. Environ Health Perspect.

[OCR_00613] Stewart I. D., Flaks B., Watanapa P., Davies P. W., Williamson R. C. (1991). Pancreatobiliary diversion enhances experimental pancreatic carcinogenesis.. Br J Cancer.

[OCR_00617] Sumi C., Longnecker D. S., Roebuck B. D., Brinck-Johnsen T. (1989). Inhibitory effects of estrogen and castration on the early stage of pancreatic carcinogenesis in Fischer rats treated with azaserine.. Cancer Res.

[OCR_00621] Townsend C. M., Singh P., Thompson J. C. (1989). Effects of gastrointestinal peptides on gastrointestinal cancer growth.. Gastroenterol Clin North Am.

[OCR_00626] Watanapa P., Efa E. F., Beardshall K., Calam J., Sarraf C. E., Alison M. R., Williamson R. C. (1991). Inhibitory effect of a cholecystokinin antagonist on the proliferative response of the pancreas to pancreatobiliary diversion.. Gut.

